# Factors associated with changes in healthy lifestyle behaviors among hematological cancer patients during the COVID-19 pandemic

**DOI:** 10.3389/fpsyg.2023.1081397

**Published:** 2023-03-09

**Authors:** Nienke Zomerdijk, Michelle I. Jongenelis, Ben Collins, Jane Turner, Camille E. Short, Andrew Smith, Kathryn Huntley

**Affiliations:** ^1^Melbourne School of Psychological Sciences, University of Melbourne, Parkville, VIC, Australia; ^2^Victorian Comprehensive Cancer Centre Alliance, Parkville, VIC, Australia; ^3^Melbourne Centre for Behaviour Change, University of Melbourne, Parkville, VIC, Australia; ^4^Faculty of Medicine, University of Queensland, Herston, QLD, Australia; ^5^Royal Brisbane and Women’s Hospital, Brisbane, QLD, Australia; ^6^School of Health Sciences, University of Melbourne, Parkville, VIC, Australia; ^7^Leukaemia Foundation, Brisbane, QLD, Australia

**Keywords:** hematology patients, cancer, diet, exercise, alcohol, COVID-19 pandemic

## Abstract

**Background:**

There is a paucity of research examining the effects of the COVID-19 pandemic on the healthy lifestyle behaviors of hematological cancer patients. We examined changes in healthy lifestyle behaviors since the pandemic and identified factors associated with these changes among members of this high-risk population.

**Methods:**

Hematological cancer patients (*n* = 394) completed a self-report online survey from July to August 2020. The survey assessed pandemic-related changes in exercise, alcohol consumption, and consumption of fruit, vegetables, and wholegrains. Information relating to several demographic, clinical, and psychological factors was also collected. Factors associated with changes in healthy lifestyle behaviors were analyzed using logistic regression.

**Results:**

Just 14% of patients surveyed reported exercising more during the pandemic (39% exercised less). Only a quarter (24%) improved their diet, while nearly half (45%) reported eating less fruit, vegetables, and wholegrains. Just over a quarter (28%) consumed less alcohol (17% consumed more alcohol). Fear of contracting COVID-19 and psychological distress were significantly associated with reduced exercise. Younger age was significantly associated with both increased alcohol consumption and increased exercise. Being a woman was significantly associated with unfavorable changes in diet and being married was significantly associated with decreased alcohol consumption.

**Conclusion:**

A substantial proportion of hematological cancer patients reported unfavorable changes in healthy lifestyle behaviors during the pandemic. Results highlight the importance of supporting healthy lifestyle practices among this particularly vulnerable group to ensure health is optimized while undergoing treatment and when in remission, particularly during crisis times like the COVID-19 pandemic.

## Introduction

The COVID-19 pandemic has created challenges that have profoundly impacted society. In countries with widespread community transmission of COVID-19, strict public health responses such as school closures, stay-at-home orders, border restrictions, and mandated self-isolation were enforced (Moss et al., 2020; Parliament of Victoria, 2022). Although the aim of these measures was to protect the population and the health system that serves them, they have disrupted all segments of the population. There is increasing recognition of the negative psychosocial consequences of these measures, including increased loneliness, reduced social support, depression, anxiety, and financial concerns (Ongaro et al., 2021). Certain subgroups of the population have been impacted more than others.

There is now considerable evidence demonstrating that hematological cancer patients are at particular risk of contracting COVID-19 and mortality from COVID-19 (Lee et al., 2020). A meta-analysis of data from 3,377 adult patients with both hematological cancer and COVID-19 reported a mortality risk of 34% (Vijenthira et al., 2020), which is significantly higher than the mortality rate reported in patients with solid tumors (22%; Zhang et al., 2021) and the average population aged 75 years (5%; Levin et al., 2020). Studies conducted during the early stages of the pandemic have shown that fears of contracting COVID-19 have contributed to heightened levels of psychological distress in hematological cancer patients (Zomerdijk et al., 2021a,b; Gates, 2022). There is also evidence that COVID-19 vaccines may yield less protection for people living with hematological cancers when compared to the general population (Lee et al., 2022; Re et al., 2022). With the progressive relaxation of public health measures, hematological cancer patients remain vulnerable to infection, highlighting the importance of ongoing COVID-19 protective behavioral measures, such as mask wearing, physical distancing, and working from home.

There is some evidence to suggest that COVID-19 related impacts have contributed to changes in healthy lifestyle behaviors, including physical activity, healthy diet, and alcohol consumption. For the general public, restrictions such as stay-at-home orders and the closure of sport facilities and public parks have contributed to a decline in exercise and fostered sedentary behavior (Wilke et al., 2021). A recent US study reported that one-third of cancer patients reduced their physical activity during the pandemic, with stressors such as disruptions to daily life being a key contributor to the decline among those surveyed (Himbert et al., 2022). Another survey study found that 17% of cancer survivors reported consuming more alcohol during the pandemic, which was associated with higher levels of anxiety and depression (Beebe-Dimmer et al., 2022).

These preliminary findings are concerning given the well documented body of evidence demonstrating the beneficial effects of healthy lifestyle behaviors on outcomes in cancer patients (Newton et al., 2020). For example, there is compelling evidence from over 30 randomized controlled trials that participating in regular exercise has benefits for preventing and managing the physical and psychosocial effects associated with cancer and its treatment (Buffart et al., 2017). These effects have also been identified in hematological cancers, with significant improvements found in treatment-related toxicities, physical functioning, and quality of life (Knips et al., 2019; Sitlinger et al., 2020). For patients undergoing planned stem cell transplantation, exercise has been shown to be beneficial for maintaining physical fitness, which is a requirement for transplant eligibility (Morishita et al., 2019). After transplantation, loss of muscle strength is highly prevalent and may cause disabilities (Morishita et al., 2013). Additionally, in the pre-and post-transplant phase, patients are hospitalized in a single-bed isolation room for several weeks, thereby aggravating the already low strength capacity induced by transplant treatment and contributing to increased fatigue (Kovalszki et al., 2008). Maintaining exercise before and after treatment is therefore key to preserving functioning in hematological cancer patients. As such, the consequences of reduced engagement in healthy lifestyle behaviors during the COVID-19 pandemic may be far reaching. However, results to date apply primarily to patients with solid tumors.

Despite their vulnerability and marked differences to patients living with solid cancers, those with hematological cancers have been largely overlooked, leaving some to call them the “Prisoners of the Pandemic” (International COVID-19 Blood Cancer Coalition, 2022). The concern for their physical and mental health was highlighted in the recent patient impact statement from the International COVID-19 Blood Cancer Coalition (2022), which calls for greater research attention to the specific impact of the pandemic on hematological cancer patients. To inform the improvement of support for those living with hematological cancers, the present study aimed to evaluate changes in healthy lifestyle behaviors among hematological cancer patients during the pandemic and the factors associated with these changes.

## Materials and methods

### Participants and recruitment

The present study formed part of a broader research program that aimed to investigate the experiences of hematological cancer patients during the COVID-19 pandemic. Participants were eligible for inclusion if they were aged ≥ 18 years, currently have, or previously have had, a confirmed diagnosis of hematological cancer, and had sufficient English language skills to participate without an interpreter. Participants who resided outside of Australia were excluded.

Participants were recruited between July and August 2020 through a study advertisement distributed *via* email and/or social media platforms by a national blood cancer community group (Leukaemia Foundation), a professional member working group (Victorian COVID-19 Cancer Network), and a clinical trial group (Australasian Leukaemia & Lymphoma Group). The advertisement included a link to an online information sheet, consent form, and survey. The University of Melbourne Human Research Ethics Committee approved the study protocol (Ref: 2057125.1).

### Measures

After providing informed consent, participants completed an online cross-sectional survey that included the items outlined below.

#### Socio-demographic characteristics

The assessed socio-demographic variables were age, gender, postcode, marital status, and education level. Residential postcode was used to classify respondents’ location (Major cities/Inner regional/Outer regional/Remote/Very remote) as per the Australian Bureau of Statistics Australian Statistical Geography Standard (Australian Bureau of Statistics, 2016). For analysis purposes, location (major cities vs. regional), marital status (single vs. married/defacto), and education (tertiary vs. non-tertiary) variables were dichotomized.

#### Fear of contracting COVID-19

A single item – ‘How concerned are you about being infected with COVID-19 yourself?’ – was created by the research team to investigate respondents’ concern about their perceived risk of contracting COVID-19. Responses were made on a 5-point scale that ranged from 1 (not at all concerned) to 5 (very concerned).

#### Psychological distress

Psychological distress was assessed using the Kessler 10-item assessment (K10; Kessler et al., 2003). Respondents indicated the extent to which they experienced various emotional states since the beginning of the pandemic using a 5-point Likert scale that ranged from 1 (none of the time) to 5 (all the time). Cronbach’s alpha in this sample was 0.92 (95% CI = 0.90–0.93), indicating excellent internal consistency. Scores were divided into three categories representing mild (range 20–24), moderate (range 25–29), and severe psychological distress (range 30–50; Kessler et al., 2003).

#### Exercise

Respondents completed the Godin Leisure-time Exercise Questionnaire (GODIN; Godin and Shephard, 1985) and were asked to estimate the frequency with which they engaged in mild (e.g., bowling, golf, easy walking), moderate (e.g., fast walking, easy swimming), and strenuous physical activity (e.g., running, vigorous swimming) over a typical 7-day period before and during the COVID-19 pandemic. Higher scores indicate higher overall activity levels.

#### Diet

Questions relating to diet, adapted from the National Health Survey (Australian Bureau of Statistics, 2018), asked respondents to report on the average number of servings of fruit, vegetables, and wholegrains consumed per day before and during the COVID-19 pandemic. A composite diet score for each respondent was calculated as the total servings of fruit, vegetables, and wholegrains consumed per day. For example, a respondent who reported consuming 2 servings of fruit, 5 of vegetables, and 3 of wholegrains would receive a composite diet score of 10.

#### Alcohol

Respondents were presented with the Australian Guidelines’ standard drink definitions (National Health and Medical Research Council, 2020) and were asked (i) how often they consumed an alcoholic drink of any kind before and during the COVID-19 pandemic and (ii) how many standard drinks (defined as 10 g of alcohol as per Australian guidelines) they usually consumed on a day that they had an alcoholic drink. Total standard drinks consumed per week was calculated for each respondent using the quantity–frequency method.

### Statistical analysis

Survey data were collected in Qualtrics and de-identified prior to being exported to R version 4.2.0 for analysis (R Core Team, 2022). One respondent did not meet age eligibility and was removed from the data set, leaving only eligible participants for the data analyses. Descriptive statistics (e.g., proportions, frequencies, means, standard deviations) were calculated for all variables of interest. Differences between pre-COVID-19 and during-COVID-19 scores were calculated for each health behavior variable (exercise, diet, alcohol consumption). Binary variables were then created to: (1) identify those who increased their engagement with each health behavior (i.e., those that had a positive difference score) versus no change or a decrease (i.e., those that had a negative or zero value for their difference score), and (2) identify those who decreased their engagement in a health behavior (i.e., those that had a negative difference score) versus no change or increase (i.e., a difference score of zero or a positive score). This resulted in a total of six dichotomized outcome variables. Predictor variables were factors potentially associated with each dichotomized health behavior variable (age, gender, location, marital status, education, fear of contracting COVID-19, psychological distress). A total of six multivariate logistic regression analyses were conducted (i.e., one for each binary outcome variable). Parameter estimates of log odds and *p*-values were examined, and a significance level of *p* < 0.05 was used to determine significance.

## Results

### Sample characteristics

A total of 394 respondents (210 men, 184 women) aged 20–84 years (*M* = 60.4, SD = 12.8) completed the survey. Respondents were included in logistic regression analyses only if they had completed all questions related to the lifestyle behavior being examined. Consequently, 308 participants were included in the exercise analysis, 330 in the diet analysis, and 232 in the alcohol consumption analysis. Chi-squared tests (for categorical variables) and *t*-tests (for continuous variables) were used to identify any demographic differences between respondents included in analyses and those with missing data. Respondents with missing exercise data were older and less likely to be tertiary educated than those included in analyses. Respondents with missing alcohol consumption data were more likely to be female than those included in analyses. No other significant demographic differences were found. Participant characteristics are summarized in Table 1.

**Table 1:** Demographic and medical characteristics of respondents by changes in healthy lifestyle behaviors (*n =* 394).

	Exercise			Diet			Alcohol consumption		
	Increase	No change	Decrease	Increase	No change	Decrease	Increase	No change	Decrease
*n*	42	145	121	78	105	147	40	128	64
Age, *M* (*SD*)	56.5 (15.4)	62.8 (10.4)	57.1 (13.2)	61.8 (12.9)	59.9 (11.7)	59.8 (13.0)	54.5 (13.6)	62.7 (11.6)	59.7 (12.0)
Gender									
Men, *n* (%)	20 (48)	66 (46)	66 (55)	46 (59)	63 (60)	69 (47)	24 (60)	72 (56)	56 (88)
Women, *n* (%)	22 (52)	79 (54)	55 (45)	32 (41)	42 (40)	78 (53)	16 (40)	56 (44)	23 (36)
Postcode									
Regional, *n* (%)	16 (38)	87 (60)	54 (45)	34 (44)	53 (50)	81 (55)	18 (45)	66 (52)	33 (52)
Metro, *n* (%)	26 (62)	56 (39)	66 (55)	44 (56)	53 (50)	63 (43)	21 (53)	60 (47)	31 (48)
Marital status									
Married, *n* (%)	26 (62)	113 (78)	86 (71)	52 (67)	74 (70)	111 (76)	28 (70)	90 (70)	55 (86)
Not married, *n* (%)	16 (38)	32 (22)	35 (29)	26 (33)	31 (30)	36 (24)	12 (30)	38 (30)	9 (14)
Education									
Tertiary, *n* (%)	21 (78)	71 (49)	71 (59)	41 (53)	52 (50)	79 (54)	21 (53)	63 (49)	41 (64)
Non-tertiary, *n* (%)	6 (14)	72 (50)	49 (41)	36 (46)	52 (50)	66 (45)	18 (45)	64 (50)	23 (36)
Primary diagnosis									
Leukaemia, *n* (%)	16 (38)	35 (24)	33 (27)	16 (21)	31 (30)	43 (29)	17 (43)	29 (23)	13 (20)
Lymphoma, *n* (%)	17 (40)	47 (32)	47 (39)	29 (37)	38 (36)	49 (33)	13 (33)	46 (36)	29 (45)
Myeloma, *n* (%)	5 (12)	33 (23)	18 (15)	14 (18)	16 (15)	32 (22)	6 (15)	28 (22)	7 (11)
Other hematological cancers^a^, *n* (%)	4 (10)	30 (21)	23 (19)	19 (24)	20 (19)	23 (16)	4 (10)	25 (20)	15 (23)
Treatments									
Stem cell transplant, *n* (%)	15 (36)	71 (49)	44 (36)	32 (41)	44 (42)	63 (43)	11 (28)	55 (43)	29 (45)
Chemotherapy, *n* (%)	38 (90)	110 (76)	99 (82)	62 (79)	89 (85)	117 (80)	31 (78)	103 (80)	54 (84)
Radiation therapy, *n* (%)	11 (26)	31 (21)	24 (20)	21 (27)	17 (16)	27 (18)	8 (20)	24 (19)	15 (23)
Other treatments^b^, *n* (%)	25 (60)	86 (59)	66 (55)	51 (65)	55 (52)	78 (53)	23 (58)	67 (52)	28 (44)
Treatment Status									
Not yet started, *n* (%)	2 (5)	11 (8)	6 (5)	7 (9)	1 (1)	11 (7)	4 (10)	11 (9)	2 (3)
Currently undergoing, *n* (%)	0 (0)	19 (13)	19 (16)	15 (19)	8 (8)	15 (10)	0 (0)	9 (7)	16 (25)
Completed and in remission, *n* (%)	22 (52)	51 (35)	40 (33)	27 (35)	46 (44)	50 (34)	17 (43)	50 (39)	18 (28)
Ongoing management, *n* (%)	13 (31)	44 (30)	38 (31)	17 (22)	35 (33)	51 (35)	17 (43)	36 (28)	22 (34)
Other, *n* (%)	5 (12)	20 (14)	18 (15)	12 (15)	15 (14)	20 (14)	2 (5)	22 (17)	6 (9)
Psychological distress, *M* (*SD*)	18.2 (6.4)	17.8 (6.1)	21.2 (7.1)	19.1 (7.2)	19.6 (7.8)	19.2 (6.2)	18.8 (5.8)	18.6 (6.7)	19.9 (7.2)
Fear of contracting COVID-19, *M* (*SD*)	3.1 (1.1)	2.8 (1.1)	3.5 (1.2)	3.1 (1.2)	3.2 (1.2)	3.1 (1.2)	3.1 (1.2)	3.1 (1.2)	3.3 (1.2)

a Other hematological cancers include myelodysplastic syndrome, myeloproliferative neoplasms, amayloidosis.

b Other treatments include targeted therapy, immunotherapy, surgery.

### Descriptive results

Most participants reported exercising less during the pandemic (39%), while 14% reported exercising more. Nearly half (45%) reported eating less fruit, vegetables, and wholegrains, while 24% improved their diet. Most participants (55%) did not change their consumption of alcohol, while 17% consumed more alcohol and 28% consumed less.

### Factors associated with lifestyle behavior changes

Table 2 presents the results from the logistic regression analyses assessing relationships between predictor variables and lifestyle behavior changes during the COVID-19 pandemic. Six significant relationships were observed. Specifically, (i) higher levels of fear of contracting COVID-19 and psychological distress were significantly associated with reduced exercise, (ii) being a woman was significantly associated with reduced consumption of fruit, vegetables, and whole grains, (iii) younger age was significantly associated with increased alcohol consumption and increased exercise, and (iv) being married was significantly associated with reduced alcohol consumption during the pandemic.

**Table 2:** Parameter estimates of log odds, measures of deviation, and *p*-values for logistic regression analyses.

	Unfavorable lifestyle behavior changes								
Predictor Variables	Exercise decrease^a^			Diet decrease^a^			Alcohol increase^a^		
	*β* [95% CI]	SE	*p*	*β* [95% CI]	SE	*p*	*β* [95% CI]	SE	*p*
*Categorical variables*									
Gender (male = 0; female = 1)	−0.08 [−0.58, 0.42]	0.26	0.77	0.54 [0.08, 0.99]	0.23	0.02	−0.12 [−0.90, 0.62]	0.39	0.75
Location (metro = 0; regional = 1)	−0.29 [−0.80, 0.21]	0.26	0.26	0.33 [−0.14, 0.80]	0.24	0.17	−0.18 [−0.97, 0.59]	0.40	0.64
Marital status (partnered = 0; single = 1)	0.11 [−0.47, 0.67]	0.29	0.71	−0.26 [−0.78, 0.25]	0.26	0.32	0.41 [−0.44, 1.21]	0.42	0.32
Education (non-tertiary = 0; tertiary = 1)	0.28 [−0.22, 0.80]	0.26	0.27	0.15 [−0.31, 0.62]	0.24	0.52	−0.10 [−0.86, 0.66]	0.39	0.79
*Continuous variables*									
Age	−0.01 [−0.03, 0.01]	0.01	0.52	−0.01 [−0.03, 0.01]	0.01	0.51	−0.05 [−0.08, −0.02]	0.02	<0.01
Psychological distress	0.05 [0.01, 0.09]	0.02	0.01	0.00 [−0.04, 0.03]	0.02	0.88	−0.03 [−0.10, 0.03]	0.03	0.33
Fear of contracting COVID-19	0.36 [0.13, 0.58]	0.11	<0.01	−0.06 [−0.26, 0.14]	0.10	0.57	−0.17 [−0.52, 0.17]	0.18	0.32
	**Favorable lifestyle behavior changes**								
**Predictor variables**	**Exercise increase** ^**b**^			**Diet increase** ^**b**^			**Alcohol decrease** ^**b**^		
	***β* [95% CI]**	**SE**	***p***	***β* [95% CI]**	**SE**	***p***	***β* [95% CI]**	**SE**	***p***
*Categorical variables*									
Gender (male = 0; female = 1)	0.26 [−0.43, 0.95]	0.35	0.46	−0.29 [−0.83, 0.24]	0.27	0.29	−0.37 [−1.02, 0.27]	0.33	0.26
Location (metro = 0; regional = 1)	−0.64 [−1.37, 0.07]	0.36	0.08	−0.33 [−0.88, 0.22]	0.28	0.24	0.16 [−0.47, 0.79]	0.32	0.62
Marital status (partnered = 0; single = 1)	0.53 [−0.21, 1.24]	0.37	0.15	0.28 [−0.31, 0.85]	0.30	0.34	−0.98 [−1.85, −0.2]	0.42	0.02
Education (non-tertiary = 0; tertiary = 1)	−0.28 [−0.98, 0.42]	0.36	0.43	−0.01 [−0.55, 0.53]	0.28	0.96	0.52 [−0.10, 1.16]	0.32	0.10
*Continuous variables*									
Age	−0.03 [−0.06, 0.00]	0.01	0.03	0.01 [−0.01, 0.04]	0.01	0.24	0.00 [−0.03, 0.03]	0.01	0.94
Psychological distress	−0.06 [−0.13, 0.00]	0.03	0.05	0.00 [−0.05, 0.04]	0.02	0.84	0.03 [−0.02, 0.07]	0.02	0.28
Fear of contracting COVID-19	−0.04 [−0.36, 0.28]	0.16	0.81	0.01 [−0.23, 0.24]	0.12	0.96	0.18 [−0.09, 0.46]	0.14	0.20

*β*, parameter estimate of log odds; CI, Confidence interval [lower, upper]; SE, Standard error; *p* < 0.05 considered significant. Each column represents a single logistic regression analysis conducted with a binary outcome variable, defined in the column header.

a Compared to exercise no change/increase, diet no change/increase, alcohol no change/decrease.

b Compared to exercise no change/decrease, diet no change/decrease, alcohol no change/increase.

## Discussion

This study sought to examine changes in healthy lifestyle behaviors among hematological cancer patients during the COVID-19 pandemic and identify factors associated with these changes. In this cross-sectional survey study, we found that a substantial proportion of the study population reported unfavorable changes in lifestyle behaviors. Specifically, more than one-third reported a decrease in the amount of exercise in which they engage and nearly half reported consuming a less healthy diet. By contrast, only a small proportion increased their exercise and consumed a healthier diet. The present study also demonstrated that a small but notable proportion of respondents reported increased alcohol use during the pandemic.

The observed reductions in healthy lifestyle behaviors are concerning as engagement in such behaviors has been shown to positively affect physical and psychosocial functioning in hematological cancer patients (Knips et al., 2019; Sitlinger et al., 2020). Furthermore, recent evidence has shown that exercise can influence survival rate in patients undergoing allogeneic stem cell transplantation (Morishita et al., 2019). As such, the reduced engagement in healthy lifestyle behaviors during the COVID-19 pandemic observed in this study may be far reaching. Our results emphasize the importance of encouraging patients to maintain healthy lifestyle practices to ensure health is optimized while they are undergoing treatment and when in remission, particularly during crisis times like the COVID-19 pandemic. The benefits of engaging in physical activity in people with cancer should be properly outlined and existing evidence-based programs for maintaining a healthy lifestyle should be promoted by healthcare providers in all settings, including primary healthcare (Buffart et al., 2017; Knips et al., 2019; Sitlinger et al., 2020).

Our results revealed that psychosocial challenges associated with the pandemic emerged as barriers to exercise participation. In particular, patients who reported higher levels of psychological distress and fears of contracting COVID-19 significantly reduced their exercise during the pandemic. Hematological cancer patients, due to their risk of developing severe illness if contracted with COVID-19, are considered a serious risk group for COVID-19 (Vijenthira et al., 2020; International COVID-19 Blood Cancer Coalition, 2022). In addition, evidence shows COVID-19 vaccination may give less effective protection for people with hematological cancer (Lee et al., 2022; Re et al., 2022). Emerging studies reported behavior changes among this population such as increased isolation to cope with fears of contracting COVID-19 (Zomerdijk et al., 2021a,b). It is possible that hematological cancer patients may have reduced their exercise due to concerns about contracting the virus in public spaces. Additionally, government-imposed public health measures included the closure of public parks and fitness facilities, which may have limited opportunities for exercise and fostered sedentary behavior.

To reduce fears of acquiring COVID-19, clinicians should adapt exercise programs for hematological cancer patients to ensure these are conducted in a safe environment (Newton et al., 2020). This is especially important in fitness facilities given coronavirus appears to exhibit strong stability on surfaces such as stainless steel that are often found in these settings (Aboubakr et al., 2021). Public awareness of the ongoing vulnerability of hematological cancer patients along with continued protective measures such as masking in exercise settings should also be encouraged, especially as public rules are being relaxed (International COVID-19 Blood Cancer Coalition, 2022). These measures could provide some reassurance for hematological cancer patients and help them to resume a normal exercise regime.

Our findings support existing evidence demonstrating significant inverse associations between physical activity levels and psychological distress (Stubbs et al., 2017; Wilke et al., 2021; Himbert et al., 2022) and suggest hematological cancer patients experiencing psychological distress are less inclined to engage in physical activity. In these patients, symptoms of psychological distress, such as lack of energy and motivation may lead to increased avoidance of physical activity (Stults-Kolehmainen and Sinha, 2014; Stubbs et al., 2017; Durosini et al., 2021). It has been reported that COVID-19 related challenges, such as concerns about treatment delays, feelings of isolation, and increased financial hardship have contributed to heightened distress in cancer patients (Zomerdijk et al., 2021a; Beebe-Dimmer et al., 2022; Gates, 2022). A recent study reported that cancer patients who felt lonely and had fewer social interactions during the pandemic were less likely to engage in exercise (Himbert et al., 2022). Taken together, these findings highlight the need to be vigilant in screening for and managing distress during crisis times like the COVID-19 pandemic, which continues to present challenges for hematological cancer patients. Clinicians can help by promoting community services that address the unique psychosocial challenges and needs within this population (Leukaemia Foundation, 2022). Additionally, group-based exercise programs can serve as a meaningful avenue for patients to develop important social relationships that can improve their psychological wellbeing as much as the physical intervention activities itself (Newton et al., 2020; Durosini et al., 2021). Remote, group-based exercise interventions can be considered for patients who remain isolated due to the risks posed by COVID-19.

Younger age emerged as a significant contributor to increased exercise among those surveyed in the present study. This finding aligns with previous studies conducted with cancer patients both pre-and during-the pandemic (Himbert et al., 2022). Younger patients advised to work or study from home may have spent less time commuting and seized the opportunity to exercise more. They may have also spent more time online and found more appropriate health related information during the pandemic than older patients. This is consistent with research reporting higher levels of digital skills and digital health literacy among younger people compared to older people (Berens et al., 2016; Thomas et al., 2018).

However, in our study, younger age was also associated with increased alcohol consumption. Indeed, while restrictions may have served as a renewed source of motivation for exercise, confinement to the home, employment changes, and lack of social connections may have also triggered increased alcohol use among young people. This is consistent with the data reported by Australia’s Foundation for Alcohol Research and Education, showing alcohol purchases during the early stages of the pandemic increased by 20%, with 33% of Australians aged under 50 years drinking more to cope with distress (Foundation for Alcohol Research and Education, 2020). Taken together, the increased exercise observed among younger patients in our study is encouraging, but the increased alcohol use is concerning. Given the established benefits of preventative health measures such as limiting alcohol consumption, additional strategies are needed to enhance awareness of the increased risk of cancer resulting from alcohol consumption (World Health Organization, 2010). These may include clinician delivered education about treatment complications associated with alcohol use (LoConte et al., 2018) and public health strategies, including educational programs highlighting the link between alcohol and cancer for young people and incorporating this messaging in school-based alcohol education programs (Bates et al., 2018).

## Limitations

There are several limitations in our study. First, the findings should be interpreted with caution due to the retrospective and self-reported nature of the data collected. Responses are subject to recall bias and causative links cannot be assumed. Longitudinal studies are needed to observe the impact of the COVID-19 pandemic on the lifestyle changes of hematological cancer patients over time. Second, our findings only illustrate the lifestyle behaviors of hematology patients within Australia during the early stages of the pandemic. The behaviors of patients in other countries are likely to differ, owing to varying degrees of COVID-19 restrictions in place at the time. Third, a number of other factors may have influenced the observed reductions in healthy lifestyle behaviors among this patient cohort. Many hematological cancer patients experience a decline in nutritional status and weight due to the side effects associated with aggressive treatments such as stem cell transplantation which can lead to reduced exercise vitality (Morishita et al., 2019).

Additionally, both patients and survivors of all hematological cancer types were included in this sample. Future studies should conduct stratified analyses by hematological cancer subtypes and treatment status. Finally, this study did not explicitly ask patients about perceived challenges in maintaining healthy lifestyle behaviors during the COVID-19 pandemic and this should be a direction of future research to inform prevention efforts. Investigating in more detail the influence of psychological factors including fears of contracting COVID-19 on physical activity would be beneficial. Future studies should expand on our results and continue to monitor health behaviors to investigate if unfavorable changes persist.

## Conclusion

The COVID-19 pandemic has had far-reaching effects on the general well-being of hematological cancer patients. Although maintaining healthy lifestyle behaviors is important for preventing and managing the physical and psychosocial effects associated with hematological cancer and its treatment, our results indicate that a significant proportion of hematological cancer patients experienced unfavorable changes in healthy lifestyle behaviors during the early stages of the pandemic. This was more common among patients who reported experiencing fear of contracting COVID-19 and increased psychological distress and patients who were younger. Supporting healthy lifestyle practices is important to ensure health is optimized while undergoing treatment and when in remission, particularly during crisis times like the COVID-19 pandemic which continues to present challenges for this group.

## Data availability statement

The raw data supporting the conclusions of this article will be made available by the authors, without undue reservation.

## Ethics statement

The studies involving human participants were reviewed and approved by The University of Melbourne Human Research Ethics Committee (Ref: 2057125.1). The patients/participants provided their written informed consent to participate in this study.

## Author contributions

NZ, MJ, BC, JT, CS, AS, and KH contributed to the study conception and design. Material preparation, data collection, and analysis were performed by NZ, MJ, and BC. The first draft of the manuscript was written by NZ. All authors contributed to the manuscript revision, read, and approved the submitted version.

## Funding

This work was supported by a private donation made to the Melbourne School of Psychological Sciences to support research into the impact of COVID-19 on hematology patients.

## Conflict of interest

The authors declare that the research was conducted in the absence of any commercial or financial relationships that could be construed as a potential conflict of interest.

## Publisher’s note

All claims expressed in this article are solely those of the authors and do not necessarily represent those of their affiliated organizations, or those of the publisher, the editors and the reviewers. Any product that may be evaluated in this article, or claim that may be made by its manufacturer, is not guaranteed or endorsed by the publisher.
